# Neurocognitive Deficits in Remitted Cases of Schizophrenia: A Tertiary Hospital-Based Cross-Sectional Study

**DOI:** 10.1192/j.eurpsy.2026.12182

**Published:** 2026-07-31

**Authors:** S. Chattopadhyay, K. Chakraborty

**Affiliations:** 1 Psychiatry, Barasat Government Medical College, Barasat, West Bengal; 2 Psychiatry, College of Medicine & JNM Hospital, Kalyani, Nadia, West Bengal, India

## Abstract

**Introduction:**

Cognitive deficits are one of the core symptoms of schizophrenia that evolve during the disease process. The cognitive domains mostly impaired are executive functions, memory, fluency, processing speed, working memory, attention, and delayed recall.

**Objectives:**

In the index study, the authors included 70 remitted patients with schizophrenia [As per the operational criteria of Remission in Schizophrenia Working Group (RSWG) (2005) & the PAN-SS scale] & assessed them with the Addenbrooke’s Cognitive Examination– version III (ACE III) and the Montreal Cognitive Assessment Test (MoCA).

**Methods:**

This cross-sectional observational study recruited 70 remitted patients (ICD-10; RSWG 2005) aged 18-50 years, who were literate in English/Hindi/Bengali, from the College of Medicine & JNM Hospital, Kalyani outpatient department (October 2022-October 2023). Patients having a history of dementia, stroke, intracranial operative procedure, autism, intellectual disability, and major neurological disorders & history of diabetes and hypertension for more than 10 years & patients having comorbid psychiatric disorders as per the Mini International Neuropsychiatric Interview (MINI)are excluded from the index study.

**Study Instruments:** Positive and negative syndrome in schizophrenia (PANSS) scale, Mini International Neuropsychiatric Interview (MINI), Addenbrooke’s Cognitive Examination-III (ACE III), Montreal Cognitive Assessment Test (MoCA). Data analysed with SPSS v22 using descriptive stats, correlations; p≤0.05 significant. Ethical clearance obtained.

**Results:**

The mean positive symptom score (15.16 ± 2.48) was higher than the mean negative symptom score (13.51 ± 2.763) (PAN-SS). Overall, the most commonly reported symptoms were delusion (positive), passive social withdrawal & lack of spontaneity (negative) & unusual thought content (general psychopathological) in the study. On ACE III, more than four-fifths (82.9%) of the subjects showed abnormal deficits and severely compromised cognitive functions. Attention, language, and total ACE-III scores did not show a difference between genders, but the memory score was found to be higher in males (13.58 ± 5.22) compared to females (12.51 ± 3.53). Females had higher fluency rates (8.2968.29 ± 1.83) & visuospatial abilities (12.18 ± 3.31) compared to males. On MOCA, the language domain was the most affected. The majority of the samples had mild impairment (51.4%) in cognitive function.

**Image 1:**
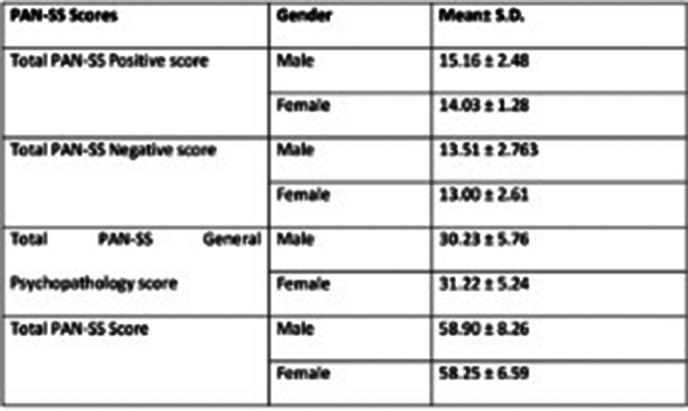
Image 1: Long description.

**Image 2:**
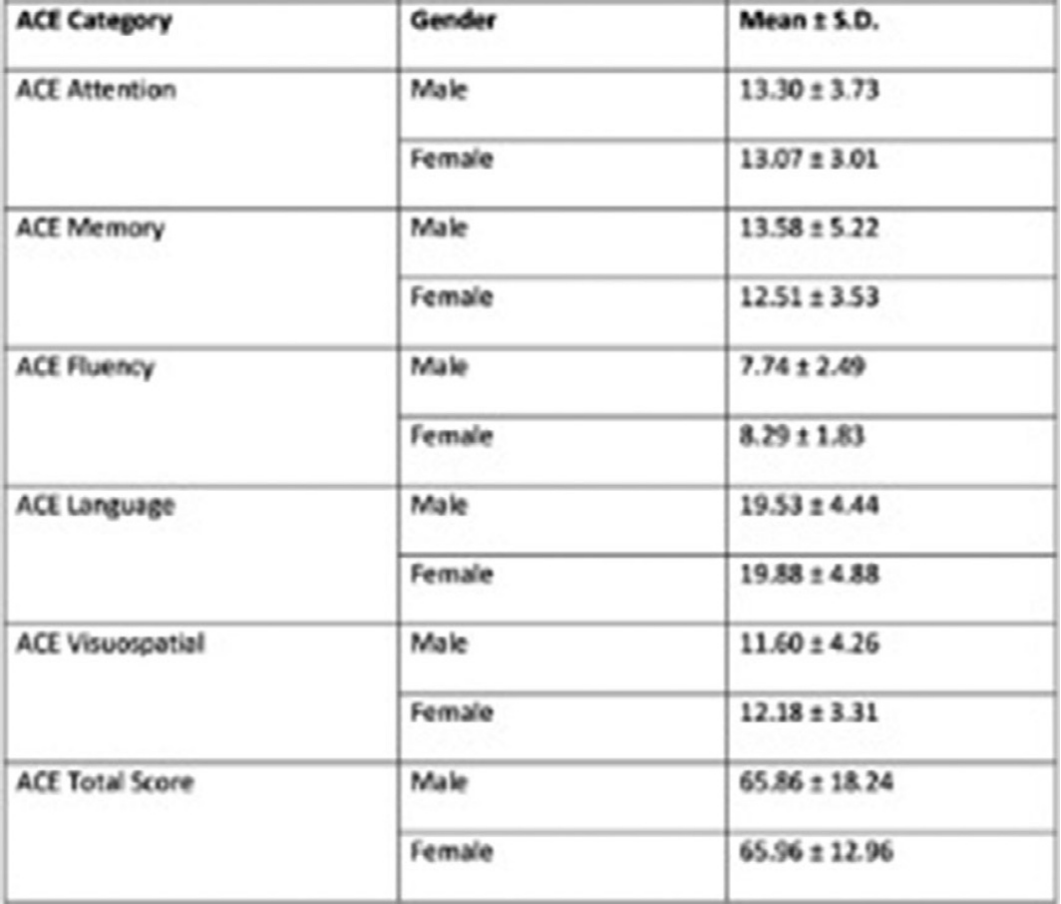
Image 2: Long description.

**Image 3:**
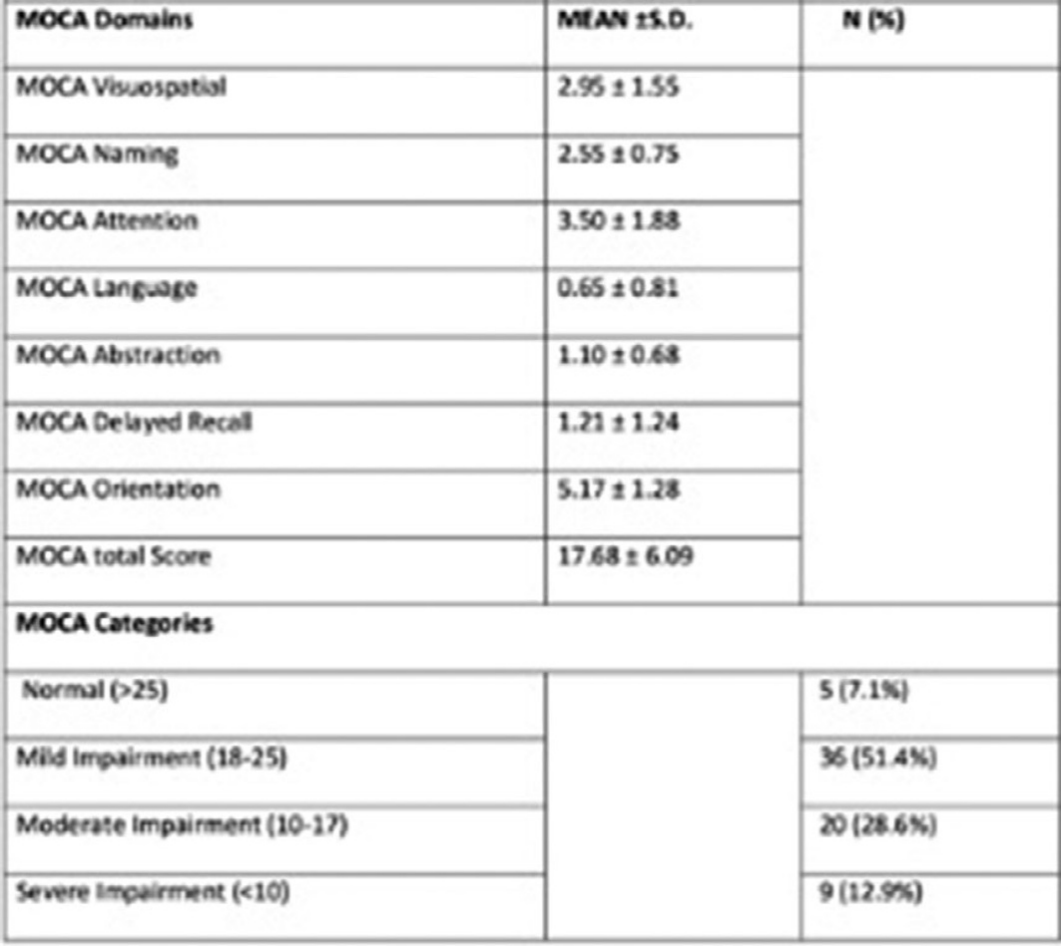
Image 3: Long description.

**Conclusions:**

The Majority of the remitted patients with schizophrenia had cognitive deficits in multiple domains, which is likely due to the disease process itself.

**Disclosure of Interest:**

None Declared

## Long descriptions

### Image 1: Long description

The table consists of three columns: P A N S S Scores, Gender, and Mean plus or minus S dot D dot.

* Total P A N S S Positive score: Male is 15.16 plus or minus 2.48. Female is 14.03 plus or minus 1.28.

* Total P A N S S Negative score: Male is 13.51 plus or minus 2.763. Female is 13.00 plus or minus 2.61.

* Total P A N S S General Psychopathology score: Male is 30.23 plus or minus 5.76. Female is 31.22 plus or minus 5.24.

* Total P A N S S Score: Male is 58.90 plus or minus 8.26. Female is 58.25 plus or minus 6.59.

Navigate back to Image 1:.

### Image 2: Long description

The table consists of three columns: A C E Category, Gender, and Mean plus or minus S dot D dot.

* A C E Attention: Male 13.30 plus or minus 3.73; Female 13.07 plus or minus 3.01.

* A C E Memory: Male 13.58 plus or minus 5.22; Female 12.51 plus or minus 3.53.

* A C E Fluency: Male 7.74 plus or minus 2.49; Female 8.29 plus or minus 1.83.

* A C E Language: Male 19.53 plus or minus 4.44; Female 19.88 plus or minus 4.88.

* A C E Visuospatial: Male 11.60 plus or minus 4.26; Female 12.18 plus or minus 3.31.

* A C E Total Score: Male 65.86 plus or minus 18.24; Female 65.96 plus or minus 12.96.

Navigate back to Image 2:.

### Image 3: Long description

The table is divided into two main sections.

The first section, titled M O C A Domains, lists mean scores plus or minus S dot D dot for eight rows:

* M O C A Visuospatial: 2.95 plus or minus 1.55

* M O C A Naming: 2.55 plus or minus 0.75

* M O C A Attention: 3.50 plus or minus 1.88

* M O C A Language: 0.65 plus or minus 0.81

* M O C A Abstraction: 1.10 plus or minus 0.68

* M O C A Delayed Recall: 1.21 plus or minus 1.24

* M O C A Orientation: 5.17 plus or minus 1.28

* M O C A total Score: 17.68 plus or minus 6.09

The second section, titled M O C A Categories, lists the count N and percentage for four impairment levels:

* Normal (greater than 25): 5 (7.1%)

* Mild Impairment (18-25): 36 (51.4%)

* Moderate Impairment (10-17): 20 (28.6%)

* Severe Impairment (less than 10): 9 (12.9%)

Navigate back to Image 3:.

